# Functionally Graded Oxide Scale on (Hf,Zr,Ti)B_2_ Coating with Exceptional Ablation Resistance Induced by Unique Ti Dissolving

**DOI:** 10.1002/advs.202411292

**Published:** 2025-01-15

**Authors:** Junshuai Lv, Wei Li, Yanqin Fu, Menglin Zhang, Lingxiang Guo, Fanyu Lu, Jiachen Li, Tao Li, Yulei Zhang, Hejun Li

**Affiliations:** ^1^ Shaanxi Key Laboratory of Fiber Reinforced Light‐Weight Composites State Key Laboratory of Solidification Processing Northwestern Polytechnical University Xi'an 710072 China; ^2^ Henan Key Laboratory of High Performance Carbon Fiber Reinforced Composites Institute of Carbon Matrix Composites Henan Academy of Sciences Zhengzhou 450046 China

**Keywords:** ablation‐resistant materials, multicomponent solid solutions, oxide scale, ultra‐high temperature ceramics

## Abstract

Multicomponent Ti‐containing ultra‐high temperature ceramics (UHTCs) have emerged as more promising ablation‐resistant materials than typical UHTCs for applications above 2000 °C. However, the underlying mechanism of Ti improving the ablation performance is still obscure. Here, (Hf,Zr,Ti)B_2_ coatings are fabricated by supersonic atmospheric plasma spraying, and the effects of Ti content on the ablation performance under an oxyacetylene flame are investigated. The (Hf_0.45_Zr_0.45_Ti_0.10_)B_2_ coating shows superior ablation resistance and cycling reliability at ≈2200°C. A functionally graded oxide scale comprising an outer dense layer and an underlying fine granular layer formed. The former is a better oxygen barrier owing to fewer cracks and the latter has high strain tolerance due to finer grain size. The uniform dissolving of ≈4 mol% Ti in the inner layer results in grain refinement via sluggish diffusion and thus stress release. For the outer layer, Ti segregation at the nanoscale leads to a metastable cubic (Hf,Zr,Ti)O_2_ and local severe lattice distortion, inhibiting the propagation of cracks. Ti ions’ unique dissolving in the oxide scale enables a strong oxygen diffusion barrier with high strain tolerance, which is responsible for superior performance. This study provides new insights into the ablation behavior of Ti‐containing multicomponent UHTCs.

## Graphical abstract

A functionally graded oxide scale comprising a dense top layer and an underlying fine‐grained layer with a grain size gradient forms on a multicomponent (Hf,Zr,Ti)B_2_ coating through Ti content optimization. The former is a superior oxygen barrier, and the latter ensures strain tolerance, collectively manifesting the coating's exceptional ablation resistance under severe thermal shock.

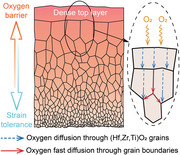

## 1 Introduction

There is an insatiable demand for materials capable of withstanding the extreme environments associated with hypersonic flight, atmosphere reentry, and rocket propulsions.^[^ ^1^, ^2^ ^]^ Carbon fiber‐reinforced carbon matrix (C/C) composites are usable in these conditions, especially at temperatures exceeding 2000 °C due to their remarkable properties, including high specific strength, superior thermal shock resistance, and great strength retention at elevated temperatures.^[^ ^3^, ^4^, ^5^ ^]^ However, C/C composites are oxidation‐sensitive in high‐temperature oxidizing environments, limiting their applications. Therefore, a range of ceramic coatings have been constructed on bare C/C composites to isolate oxidants and heat flows.^[^ ^6^ ^]^ Ultra‐high temperature ceramic (UHTC) borides (typically HfB_2_ and ZrB_2_) have been used as coating materials because of their high melting points above 3000 °C, strong thermochemical stability, and relatively good oxidation resistance.^[^ ^7^, ^8^ ^]^ The oxidation behavior of UHTCs depends on the features of the resulting oxide scale acting as an oxygen diffusion barrier. Unfortunately, monolithic UHTC borides oxidized to form oxide scales with massive open and interconnected pores at ultra‐high temperatures (>1800 °C).^[^ ^9^ ^]^ Oxygen permeates inward through these channels, further oxidizing the underlying substrates rapidly. Given this, SiC^[^ ^10^, ^11^ ^]^ or/and transition metal silicides (typically MoSi_2_,^[^ ^12^ ^]^ TaSi_2_,^[^ ^13^ ^]^ and WSi_2_^[^ ^14^ ^]^) have been incorporated into UHTC borides to form silica, sealing the pores in the oxide scale. However, the active oxidation of SiC dominates at ultra‐high temperatures, resulting in a non‐protective gaseous oxidation product, SiO.^[^ ^15^ ^]^ Additionally, the oxide scale composed of borosilicate and refractory oxides tends to be peeled off under high‐velocity scouring due to its reduced viscosity at ultra‐high temperatures.^[^ ^16^ ^]^ A ZrB_2_‐SiC coating exposed to an oxyacetylene flame with a heat flux of 2.4 MW m^−2^ for only 60 s showed a linear recession rate of up to 0.6 µm s^−1^.^[^ ^17^ ^]^ At present, boride and silicide‐modified boride coatings still struggle to protect C/C composites at ultra‐high temperatures above 2000 °C, and therefore UHTC systems that produce oxide scales with low oxygen diffusivity and high thermal stability have been pursued.

High‐entropy UHTC borides, a class of multicomponent solid solution ceramics, generally containing four, five, or even more metal elements in equimolar, have emerged as a hotpot in the ceramic community.^[^ ^18^, ^19^, ^20^, ^21^ ^]^ They are attractive in thermal protection applications due to their favorable properties, especially better oxidation resistance than most individual borides.^[^ ^22^ ^]^ The current studies on high‐entropy UHTCs generally attribute the improved oxidation resistance to the synergistic effects of oxidation products.^[^ ^23^, ^24^, ^25^ ^]^ However, this mechanism is obscure, and in some cases, the contribution of certain elements to ablation resistance is very probably negative. Our previous work on a high‐entropy (Hf,Zr,Ta,Ti)B_2_ coating reveals that excessive liquid formation at high temperatures resulting from Ta and Ti is detrimental to the structural integrity of the oxide scale and thus ablation resistance.^[^ ^26^ ^]^ Therefore, the transition from equimolar to non‐equimolar is essential for improved ablation resistance of multicomponent UHTCs. Although the mixing entropy is not maximal in this case, an expanded composition space is available. A series of non‐equimolar multicomponent UHTC systems, including Hf‐Zr‐Ti, Hf‐Zr‐Ti‐Nb, and Hf‐Zr‐Ti‐Ta, was developed by Ye et al.,^[^ ^27^, ^28^ ^]^ and the Hf_0.5_Zr_0.3_Ti_0.1_Ta_0.1_C demonstrates a remarkable improvement in ablation resistance. We also reduced the Ta proportion in multicomponent (Hf,Zr,Ta)B_2_ coatings, resulting in superior ablation performance.^[^ ^29^ ^]^ Based on the above, non‐equimolar multicomponent UHTCs are more promising for breakthroughs in ablation resistance than general high‐entropy UHTCs.

Among the multicomponent UHTC systems, Hf and Zr typically act as the basis thanks to their oxide scales’ thermal stability. Ti‐containing Hf/Zr‐based systems are attractive in ablation‐resistant coating applications because Ti‐related oxides positively contribute to the oxide scales.^[^ ^30^, ^31^, ^32^ ^]^ For example, the self‐sealing effect induced by Ti doping results in a dense Zr_0.80_Ti_0.20_O_2_ scale on a Zr_0.8_Ti_0.2_C_0.74_B_0.26_‐based composite material.^[^ ^31^ ^]^ Furthermore, the decomposition pressure of TiO_2_ is lower than that of SiO_2_, the typical self‐healing product, favoring the oxide scale integrity.^[^ ^33^ ^]^ However, excessive Ti is unfavorable because of the relatively low melting points of Ti‐oxides.^[^ ^34^ ^]^ Therefore, the effect of Ti content for the Hf‐Zr‐Ti system on the ablation resistance is of great significance. Nevertheless, to the best of our knowledge, few studies have focused on this issue, and thus their potential exceptional performance has not been realized. In addition, the ablation behavior of the Hf‐Zr‐Ti UHTC bulk and coatings have not been thoroughly recognized at the multi‐scale, which is crucial for elucidating synergistic effects and extending to multicomponent UHTCs containing more elements to enhance ablation resistance.

In this work, we designed multicomponent (Hf,Zr,Ti)B_2_ coatings starting from a (Hf_0.5_Zr_0.5_)B_2_ base and introducing Ti into Hf/Zr sites, with nominal compositions of (Hf_0.50‐_ *_x_*_/2_Zr_0.50‐_ *_x_*_/2_Ti*_x_*)B_2_ (*x* = 0, 0.10, and 0.20). The coatings were fabricated on C/C composites by supersonic atmospheric plasma spraying (SAPS) and their ablation resistance and cycling reliability were assessed using an oxyacetylene flame. A thorough multi‐scale analysis of the resulting oxide scales reveals Ti's positive contribution to the ablation resistance, originating from the unique dissolving of Ti ions in the oxide scale. This study provides new insights into the ablation behavior of Ti‐containing UHTCs and guidance for further optimization of emerging multicomponent UHTCs for ablation‐resistant applications.

## 2 Results and Discussion

### 2.1 Preparation, Microstructure, and Composition of the Coatings

**Figure 1:**
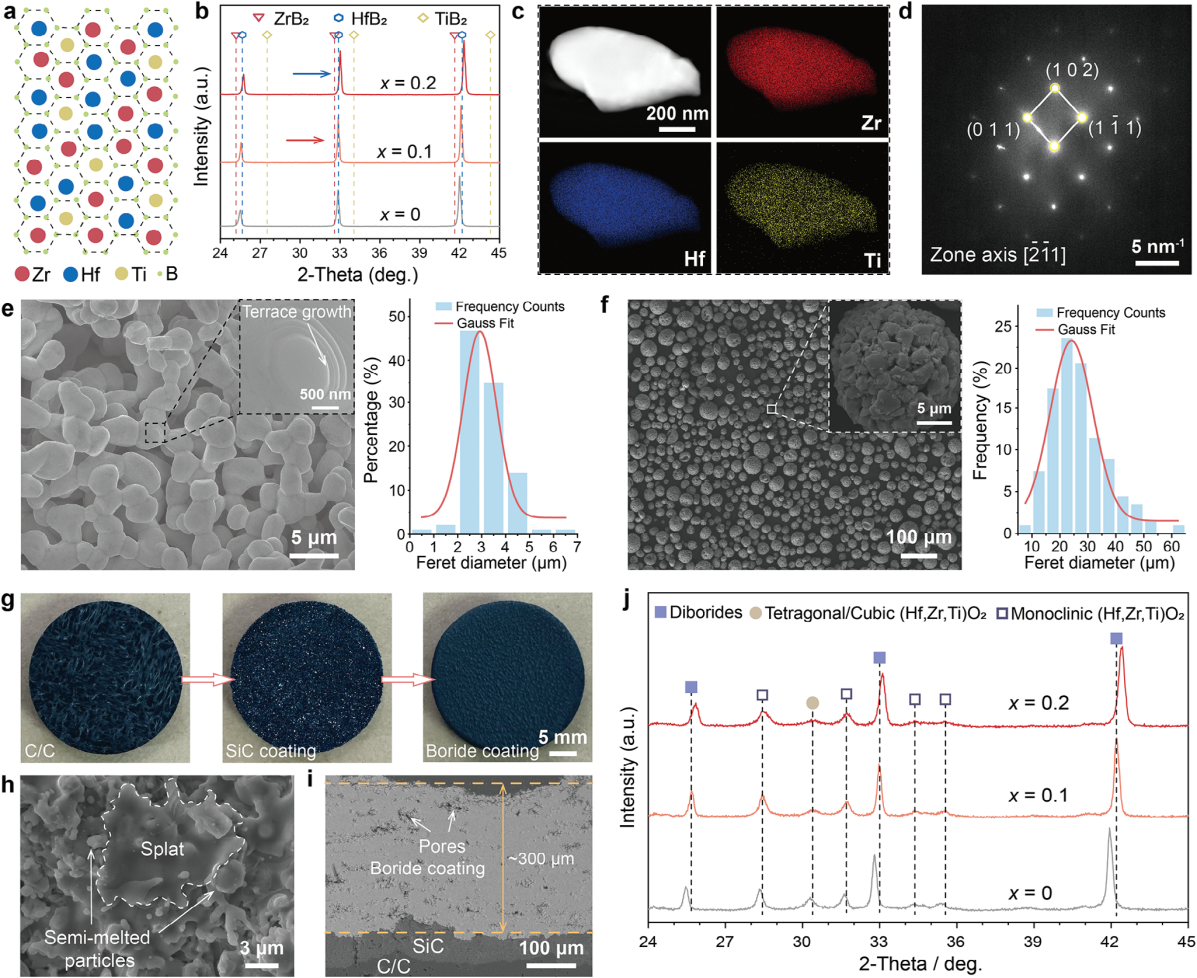
Phase composition and microstructure of the multicomponent (Hf,Zr,Ti)B_2_ powders and coatings. a) Schematic lattice structure of (Hf,Zr,Ti)B_2_ solid solution, b) X‐ray diffraction (XRD) patterns of the as‐synthesized powders. c) High‐angle annular dark‐field (HAADF) image with (energy dispersive spectroscopy) EDS elemental maps of Hf, Zr, and Ti. d) Selected area electron diffraction (SAED) pattern for a grain with *x* = 0.1. e) Scanning electron microscopy (SEM) images of the *x* = 0.1 powder together with particle size distribution. The magnified image in the inset shows the terrace growth of the boride. f) SEM image of the spray feedstock (*x* = 0.1) with particle size distribution. g) Photographs of the C/C substrate without coating, with SiC and boride coating. h,i) Surface and cross‐sectional SEM image of the *x* = 0.1 coating. j) XRD patterns of the sprayed coatings.

We employed a multicomponent solid solution strategy to achieve a homogenous distribution of Zr, Hf, and Ti at the atomic level, as illustrated in **Figure** **1**a, and therefore potential weak phases are avoided compared to heterogeneous modified UHTCs. The powders with nominal compositions of (Hf_0.5‐_ *_x_*_/2_Zr_0.5‐_ *_x_*_/2_Ti*_x_*)B_2_ (*x* = 0, 0.1, and 0.2) were synthesized by boro‐carbothermal reduction of transition metal oxides (HfO_2_, ZrO_2_, and TiO_2_) at 1800 °C. The atomic content at the cation sites was controlled by adjusting the ratio between the oxides in the raw materials. The XRD patterns of the as‐synthesized powders (Figure 1b) match the expected single‐phase solid solutions with AlB_2_‐type crystalline. The XRD peaks shift toward the high angle with increasing Ti proportion, since Ti with a smaller cation radius (0.61 Å) substituting Hf and Zr with larger cation radii (Hf: 0.71 Å; Zr: 0.72 Å) resulted in lattice contraction. Taking a grain with *x* = 0.1 as an example (Figure 1c), the corresponding SAED pattern (Figure 1d) confirms the hexagonal AlB_2_‐type crystal structure. The EDS mapping (Figure 1c) demonstrates the uniform distribution of Hf, Zr, and Ti. The above results indicate that we successfully synthesized the multicomponent powders with nominal compositions of (Hf_0.5‐_ *_x_*_/2_Zr_0.5‐_ *_x_*_/2_Ti*_x_*)B_2_ (*x* = 0, 0.1, and 0.2). Figure 1e shows the SEM image of the *x* = 0.2 powder. The corresponding particle size distribution demonstrates an average ferret diameter of ≈3 µm. The enlargement in the inset reveals the terrace growth of the borides. The prepared powders were agglomerated to provide appropriate flowability of the feedstock for efficient coating deposition. As shown in Figure 1f, the accumulated boride powder is round with an average size of ≈25 µm, which is suitable for SAPS.

The optical images (Figure 1g) show a SiC transition layer was first fabricated on the C/C substrate to alleviate the thermal expansion mismatch between the C/C substrate and the boride coating. Subsequently, the boride feedstock was heated and accelerated, impacting the SiC‐coated C/C substrate. The stacking of the splats developed in a relatively dense coating, as shown in the surface and cross‐sectional image of the *x* = 0.1 coating (Figure 1h,i). The average thickness of the three coatings was controlled to be ≈300 µm (Figure S1a–c, Supporting Information). A few pores existed due to incomplete melting of the boride particles during the SAPS process. The sprayed boride layers are interlocked with the SiC inner layers, imparting the coatings’ desirable bonding strengths of ≈6.5 MPa (see Figure S2, Supporting Information for more details). The surface XRD patterns of the sprayed coatings (Figure 1j) indicate that the major phases are boride solid solutions. As the Ti proportion increases, the shift of the boride‐associated peaks toward the high angle is still visible, indicating the difference in the Ti content of the borides. The EDS mapping results of the sprayed coating (Figure S1d,e, Supporting Information) demonstrate the uniform elemental distribution. Monoclinic (m‐), tetragonal (t‐), and/or cubic (c‐) oxides were also detected because the borides were oxidized during the oxygen‐containing spraying process. The metastable t‐ and/or c‐oxide existed because SAPS is a non‐equilibrium process.^[^ ^35^ ^]^ The oxides in the *x* = 0 coating were determined as (Hf,Zr)O_2_ owing to the complete solubility of HfO_2_ and ZrO_2_.^[^ ^36^ ^]^ Furthermore, the peaks associated with the oxides in the Ti‐containing coating slightly shifted to the high angle relative to (Hf,Zr)O_2_ due to Ti‐ions substituting on Hf and Zr sites, and thus the oxides can be identified as (Hf,Zr,Ti)O_2_. The coating's ablation resistance highly depends on its oxide scale. These oxides will also participate in the oxide scales and play a role in ablation resistance under extreme environments.

### 2.2 Ablation Resistance of the Coatings

**Figure 2:**
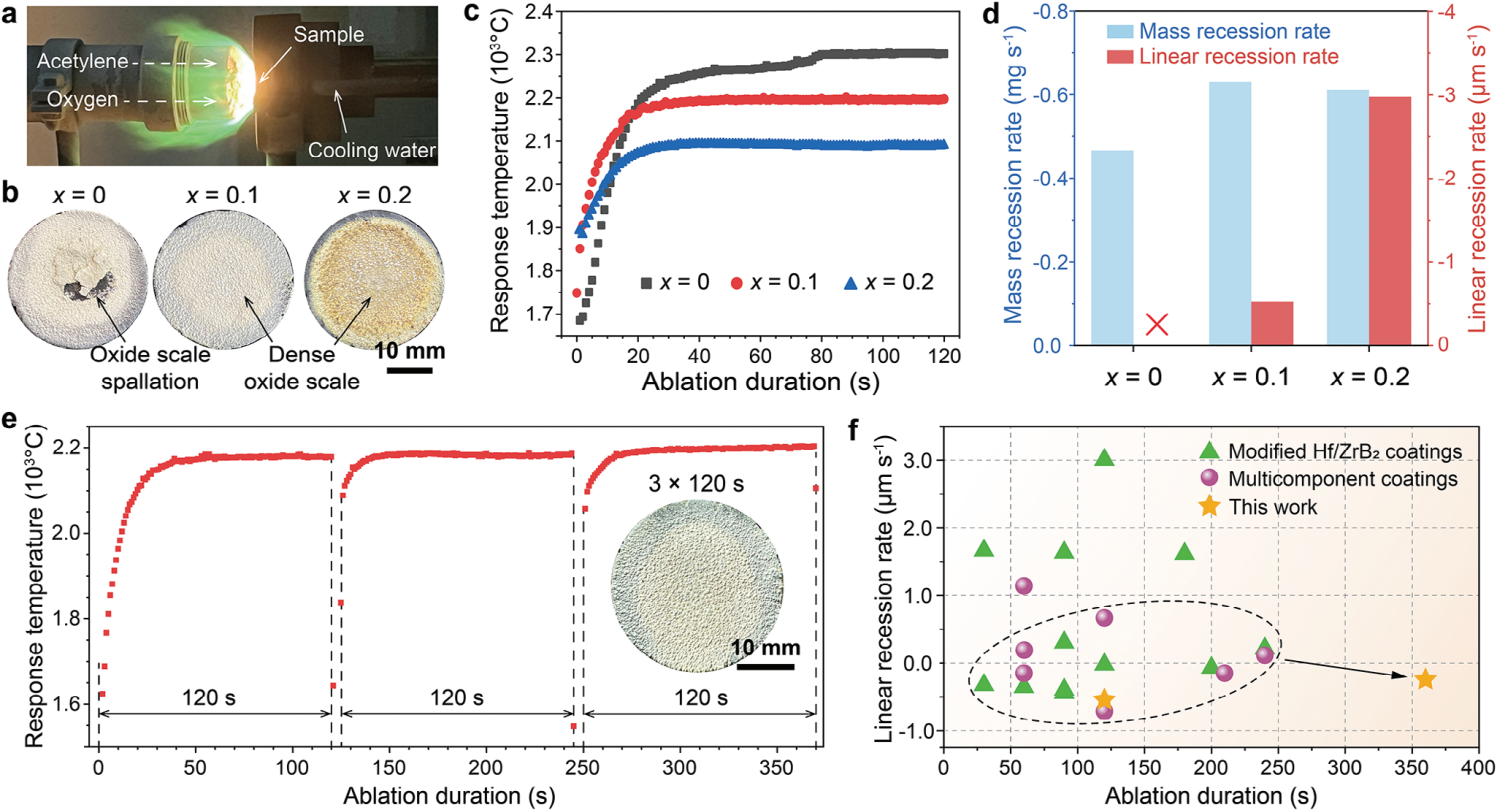
Ablation performance of the multicomponent coatings. a) Photograph of the oxyacetylene torch testing. b) Comparison of the surface morphology of the ablated coatings. c) Surface center response temperature of the samples during 120‐s ablation. d) Mass/linear recession rates of the samples after 120‐s ablation. e) Surface center response temperature of the sample with *x* = 0.1 coating during cyclic ablation testing and surface morphology after the testing. f) Comparison of the linear recession rate of the *x* = 0.1 coating with a range of representative modified boride coatings and multicomponent UHTC coatings. All coatings listed were tested using the oxyacetylene torch with a 2.4 MW m^−2^ heat flux.

An oxyacetylene torch with a heat flux of 2.4 MW m^−2^ was used to simulate the extreme environments encountered by C/C composites in aerospace applications (**Figure** **2**a). Optical images of the samples after 120‐s ablation tests (Figure 2b) demonstrate oxide scales formed, which played a key role in impeding oxygen diffusion inward and defending the flame scour. The *x* = 0 oxide scale was delaminated and destroyed, but the Ti‐containing coatings’ scales maintained structural integrity. The surface center response temperature of the samples increased parabolically during the ablation testing and stabilized at various temperatures (Figure 2c). The stable temperature decreasing with increasing Ti proportion is associated with the Ti‐related oxides with lower melting points. Additionally, the surface response temperature of the *x* = 0 coating slightly increased at 70 s, related to the local stagnation point ablation triggered by the protruding oxide fragments. The ablation performance of the samples was evaluated by mass recession rate (*R*_m_) and linear recession rate (*R*_l_) (Figure 2d). The negative values represent the increase in mass and thickness of the samples. The samples’ mass increased after 120‐s ablation as the oxide scale grew to offset the release of gaseous CO and B_2_O_3_. The *x* = 0.2 sample shows a much higher linear increase (−2.97 µm s^−1^) than the *x* = 0.1 sample (−0.53 µm s^−1^). The large thickness increase implies excessive oxide scale growth, which is generally unfavorable. A slight thickness increase of the *x* = 0.1 coating is ideal, indicating its considerable oxidation resistance.

Furthermore, cycling reliability is also a significant consideration for applications. The preferred *x* = 0.1 coating still shows negative final recession rates (*R*_m_ = −0.18 mg s^−1^; *R*_l_ = −0.23 µm s^−1^) after three 120‐s ablation cycles, with a stable response temperature of ≈2200 °C (Figure 2e). There are no visible ablation pits and cracks in the resulting oxide scale (see the inset in Figure 2e), implying the great cycling reliability of the *x* = 0.1 coating. Figure 2f summarizes *R*_l_ versus ablation duration for the *x* = 0.1 coating and other coatings reported previously; the coatings tested by the oxyacetylene torch with a 2.4 MW m^−2^ heat flux were listed to allow comparison. The (Hf,Zr,Ti)B_2_ coating with 10 mol% Ti addition shows significant improvement in ablation performance compared with individual boride coating, silicide/carbide/oxide‐modified boride coatings, and emerging multicomponent UHTC coatings (see Table S1, Supporting Information for more details).

### 2.3 Ablation Mechanism of the Coatings

**Figure 3:**
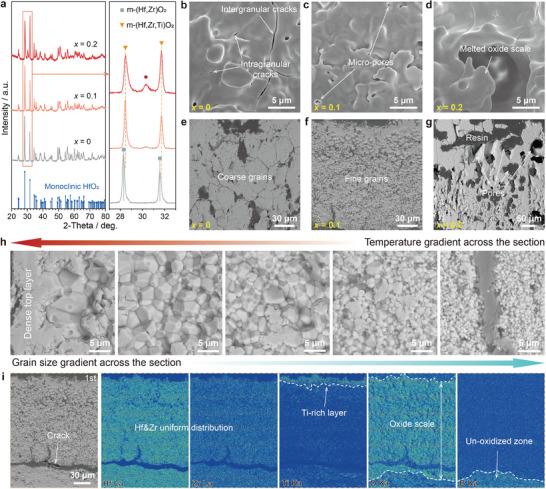
Characterization of the resulting oxide scales on the (Hf_1‐_ *_x_*_/2_Zr_1‐_ *_x_*_/2_Ti*_x_*)B_2_ coatings after the 120‐s ablation tests. a) XRD patterns on the surface center of the samples. b–d) Surface SEM images of the oxide scales on the coatings. e–g) Cross‐sectional SEM images of the oxide scales on the coatings. h) Magnified cross‐sectional SEM images of the oxide scale on the *x* = 0.1 coating show a dense top layer and an underlying fine‐grained layer with a grain size gradient across the section. i) Overviews of the *x* = 0.1 oxide scales with electron probe microanalysis elemental maps.

To clarify the improved mechanism of the ablation performance of the *x* = 0.1 coating, the composition and microstructure of the resulting oxide scales were characterized. **Figure** **3**a shows surface XRD patterns on the central region of the oxide scales. The major phase of the three oxide scales is m‐oxide. Notably, the peaks associated with the m‐oxides of the Ti‐containing coatings shift toward higher 2‐theta relative to the corresponding peaks of the ablated (Hf,Zr)B_2_ coating, implying lattice contraction. It results from Ti^4+^ dissolving into (Hf,Zr)O_2_ and therefore these m‐oxides were identified as (Hf,Zr,Ti)O_2_. In addition, a minor phase in the Ti‐containing oxide scales appeared, with the strongest peak at ≈30.3° and the intensity increasing with the increase of Ti proportion. The minor phase still existed in the *x* = 0.1 coating's oxide scale formed after three 120‐s ablation cycles (Figure S3, Supporting Information). Potential Ti‐containing phases, including t‐(Hf,Zr,Ti)O_2_, c‐(Hf,Zr,Ti)O_2_, and orthorhombic‐(Hf,Zr)TiO_4_, are consistent with the strongest peak. However, it could not be identified due to the diffraction peak overlapping. It has been determined in conjunction with subsequent TEM characterization.

Figure 3b–g and Figure S4 (Supporting Information) show the microstructure of the center heat‐affected area of the coatings upon 120‐s ablation. The (Hf,Zr)O_2_ scale was delaminated and cracked, exposing the fresh coating (Figure S4a,d, Supporting Information). The magnified surface image (Figure 3b) shows that not only intergranular cracks but also intragranular cracks are present. According to the HfO_2_‐ZrO_2_ pseudo‐binary phase diagram,^[^ ^37^ ^]^ during the cooling stage of the ablation test, (Hf_0.5_Zr_0.5_)O_2_ transformed from t‐ to m‐phase at ≈1437°C, accompanied by a volume expansion of ≈5%.^[^ ^38^ ^]^ Therefore, even intragranular cracks exist due to the tensile stress induced by the volume expansion. In contrast, the *x* = 0.1 oxide scale is intact and still adheres to the unoxidized coating (Figure 3c; Figure S4b,e, Supporting Information). However, more Ti incorporation is unsatisfactory. The *x* = 0.2 oxide scale shows characteristics of melting and recrystallization in which numerous holes exist (Figure 3d,g; Figure S4c,f, Supporting Information). The holes originated from gaseous oxidation products (B_2_O_3_ and CO) and are also considered oxygen rapid transport channels. Therefore, the *x* = 0.2 coating was consumed completely, and the resulting oxide scale was delaminated from the SiC transition layer after 120‐s ablation. The state of the oxide scale at high temperatures is responsible for its oxygen barrier properties. The high‐temperature phase composition of the *x* = 0.1 oxide scale is solid t‐(Hf,Zr)O_2_, but for Ti‐containing oxide scales, it is dominated by t‐(Hf,Zr,Ti)O_2_ solid solution, based on the thermodynamic analysis (Figure S5, Supporting Information) combined with ZrO_2_‐TiO_2_^[^ ^39^ ^]^ and HfO_2_‐TiO_2_^[^ ^40^ ^]^ phase diagrams. Additionally, more Ti‐rich liquid exists in the *x* = 0.2 oxide scale during ablation owing to the limited solubility of Ti^4+^ in (Hf,Zr,Ti)O_2_ (excess Ti ions exist in the form of liquid phase). The higher the volume fraction of liquid in the oxide scale, the lower the viscosity of the oxide scale.^[^ ^41^ ^]^ Accordingly, the *x* = 0.2 oxide scale has the lowest viscosity during ablation because of the excessive Ti doping. Viscosity is negatively related to oxygen diffusivity according to the Stokes‐Einstein relationship,^[^ ^42^ ^]^ and thus massive undesirable large bubbles exist in the *x* = 0.2 oxide scale (Figure 3g). Similar porous oxide scale cross‐sections were also reported in ablated Zr_0.8_Ti_0.2_C_0.74_B_0.26_‐dominated composites,^[^ ^31^ ^]^ whereas our coating with 10 mol% Ti doping shows a dense oxide scale, considerably slowing the oxidizing process.

The appropriate Ti doping amount not only alleviated the oxidation but also, interestingly, the oxide grains of the *x* = 0.1 oxide scale are equiaxed and much finer than those of the other two coatings, as apparent in the magnified cross‐sectional images (Figure 3e–g). It is known that plastic deformation of polycrystalline solids occurs by grain boundary sliding.^[^ ^43^, ^44^ ^]^ The fine equiaxed grains ensure deformation under thermal stress without catastrophic fracture,^[^ ^45^, ^46^ ^]^ as presented by the *x* = 0.1 oxide scale. In contrast, the *x* = 0, 0.2 oxide scales composed of coarse grains were delaminated from the substrates and damaged. The phenomenon of grain refinement induced by appropriate Ti doping is unique compared with the reported Ti‐containing multicomponent coatings/composites.^[^ ^27^, ^28^, ^30^, ^31^ ^]^ They generally formed oxide scales with coarse grains because excessive liquid induced by more Ti, Ta, Nb, and Si elements accelerated mass transfer during ablation. Similarly, the oxide grains of the *x* = 0.2 scale coarsened through the Ti‐rich liquid, resulting in reduced grain boundaries (GBs) and oxide scale delamination.

The challenge created by the high GB density is the increase in oxygen diffusivity because the oxygen diffusion rate is generally higher at the GBs than through oxide grains. Notably, the magnified cross‐sectional SEM image of the *x* = 0.1 oxide scale (Figure 3h) reveals a dense top layer and an underlying fine grains layer with a grain size gradient associated with the temperature gradient across the section during ablation. The top layer is almost free of GBs and plays a more significant role in inhibiting oxygen penetration than the underlying layer, which has a much higher GB density. This is the cause of the lightest oxidation depth of the *x* = 0.1 coating. The grain size gradient remained after exposure to prolonged high temperatures (see the cross‐section after the 3^rd^ ablation cycle; Figure S6, Supporting Information). Although the grains near the surface grow, those around the oxidizing interface are still finer than those of the *x* = 0 and *x* = 0.20 coatings tested for only 120 s. They enable stress release through grain boundary slipping and cracking, thus maintaining the structural integrity of the *x* = 0.1 oxide scale after the 3^rd^ ablation cycle.

Figure 3i shows the elemental distribution across the *x* = 0.1 oxide scale after the 1^st^ ablation cycle. Hf and Zr are uniformly distributed, but Ti is especially segregated in the outermost part of the oxide scale. The oxide scale can be divided into Ti‐rich and Ti‐poor layers corresponding to the dense top layer and the equiaxed grain layer. The Ti‐rich layer formed due to the migration outward of Ti ions, which has been identified in studies on the oxidation behavior of Ti‐containing ceramics.^[^ ^47^, ^48^, ^49^ ^]^ It was demonstrated that point defects including interstitial Ti ions and O‐ion vacancies exist in the Ti‐oxide structure and accelerate the mobility of Ti ions. Additionally, according to the Δ*G* calculations of the potential oxidation reactions (Figure S7, Supporting Information), TiO is a more thermodynamically favorable oxidation product than TiO_2_ within a broad temperature range in the Ti‐O system, consistent with the calculations conducted by Backman et al.^[^ ^50^ ^]^ However, it was rarely observed in the Ti‐compounds’ oxide scales because it has high mobility and fast transforms to TiO_2_ at a higher O_2_ partial pressure.^[^ ^51^, ^52^ ^]^ This process probably drove the Ti ion diffusion to the surface. The cross‐sectional SEM image of the *x* = 0.1 coating after the 3^rd^ ablation cycle with elemental maps (Figure S8, Supporting Information) shows that the top dense layer corresponding to the Ti‐rich layer still exists, and its average thickness increased from 12 µm to 39 µm. The oxide scale also grew from ≈170 µm after the 1^st^ cycle to ≈328 µm after the 3^rd^ cycle. To obtain the oxidation kinetics, the oxide growth rate constant was calculated based on the measured scale thicknesses, assuming *X* ^2^ = *k*_p_ *t*, where *X* represents the scale thickness in millimeters, *t* is the duration in hours, and *k*_p_ is the parabolic rate constant in mm^2^ h^−1^. The parabolic rate constant of the *x* = 0.1 coating after linear fitting (Figure S9, Supporting Information) is 1.06 ± 0.04 mm^2^ h^−1^, slightly lower than the reported typical UHTC systems (HfB_2_‐SiC, HfC, HfC‐TaC, etc.) at 2200 °C.^[^ ^42^ ^]^ This implies the diffusion‐controlled oxidation and the oxide scale's considerable oxygen barrier property. In addition, as shown in Figure 3i, a horizontal crack at the oxidizing interface formed at the cooling stage because of the thermal expansion mismatch between the oxide scale and the original coating. A crack also existed at a corresponding position in the coating after the 3^rd^ ablation cycle (see Figure S8, Supporting Information), while the cracks potentially formed in the previous cooling stages disappeared. They were probably sealed in the subsequent ablation cycle through grain coarsening and Ti ions out‐diffusion processes. The self‐sealing effect of the coating is also favorable for reusable applications.

**Figure 4:**
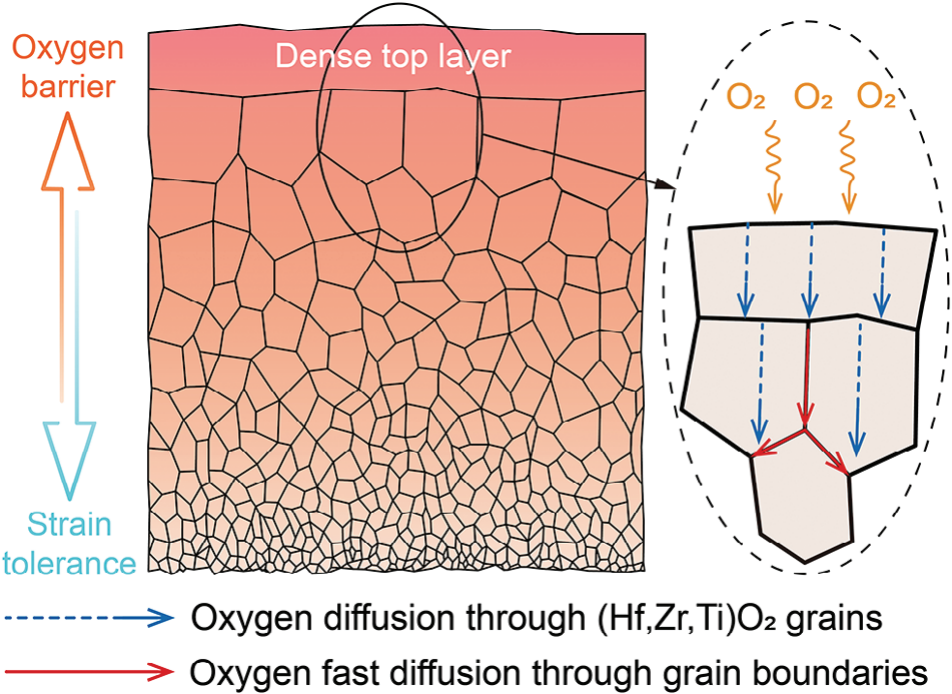
Schematic representation of the unique structure of the functionally graded oxide scale on the *x* = 0.1 coating and oxygen diffusion through the oxide scale.

**Figure** **4** illustrates the unique architecture of the resulting oxide scale on the *x* = 0.1 coating. The oxide scale consists of the dense top layer and the underlying fine‐grained layer with the grain size gradient. The former is a superior oxygen barrier due to few GBs, and the latter induced stress release through extensive GB sliding, collectively manifesting as oxidation resistance and strain‐tolerance functions. The functionally graded oxide scale mitigated coating consumption and ensured structural integrity, enabling exceptional ablation resistance under severe thermal shock.

**Figure 5:**
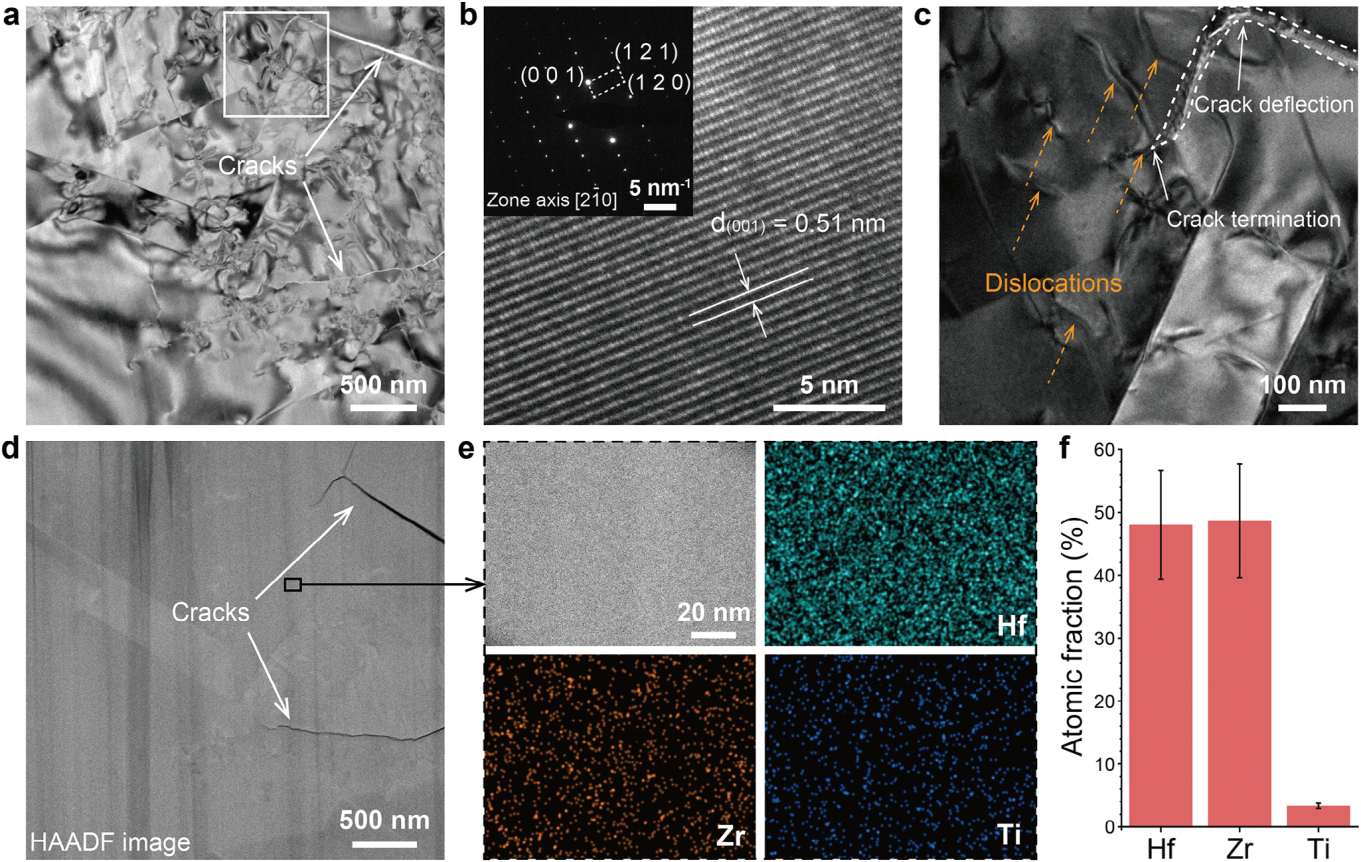
Transmission electron microscopy (TEM) analysis of the inner oxide scale (Ti‐poor layer) of the *x* = 0.1 coating after the 3^rd^ ablation cycle. a) Bright‐field (BF) TEM image showing a defective structure. b) High‐resolution (HR) TEM image and SAED pattern. c) Magnified TEM image showing dislocation‐induced crack deflection and thus termination. d,e) HAADF images and elemental maps of Hf, Zr, and Ti. f) EDS quantification corresponding to (e).

To further recognize the top and fine‐grained layers, two oxide foils were taken from the corresponding layers of the *x* = 0.1 oxide scale formed after three ablation cycles using a focus ion beam. **Figure** **5** shows the TEM characterization of the foil lift from the equiaxed grain layer. As shown in the bright‐field (BF) image (Figure 5a), numerous dislocations and cracks are present. The high‐resolution transmission electron microscopy (HR‐TEM) image and corresponding SAED pattern (Figure 5b) reveals the common m‐phase structure, consistent with the main phase detected in the XRD results (Figure 3a). The defective structure is a typical m‐(Zr/Hf)O_2_ feature because of the t‐to‐m transformation‐induced crystalline deformation. Interestingly, as shown in the magnified image (Figure 5c), a crack was deflected and thus terminated by the interaction with the dislocations. Han et al.^[^ ^53^ ^]^ reported that the strain field around dislocations led to crack deflection and bridging, which increased the fracture toughness by ≈70%. Inducing massive dislocations by phase transformation appears to be an approach for toughening ceramics. In turn, phase transformation is considered the origin of catastrophic failure. Additionally, this mechanism is not unique to the *x* = 0.1 coating and therefore is not responsible for its superior performance.

The HAADF image (Figure 5d), a Z‐contrast image, corresponding to the BF image (Figure 5a), shows no obvious contrast, implying an even elemental distribution. Also, the EDS mapping (Figure 5e) demonstrates no elemental segregation at the nanoscale. The corresponding quantification (Figure 5f) reveals that the solubility of Ti in the fine (Hf,Zr,Ti)O_2_ grains is ≈4%. The Ti ion dissolving rendered that the inner (Hf,Zr,Ti)O_2_ grains have a much finer average size than that of the (Hf,Zr)O_2_ grains, even after three ablation cycles (Figure S6, Supporting Information). The deep traps induced by Ti^4+^ in the (Hf,Zr,Ti)O_2_ solid solution greatly hinder volume diffusion in the coarsening process at elevated temperatures, resulting in grain refinement.^[^ ^54^ ^]^

**Figure 6:**
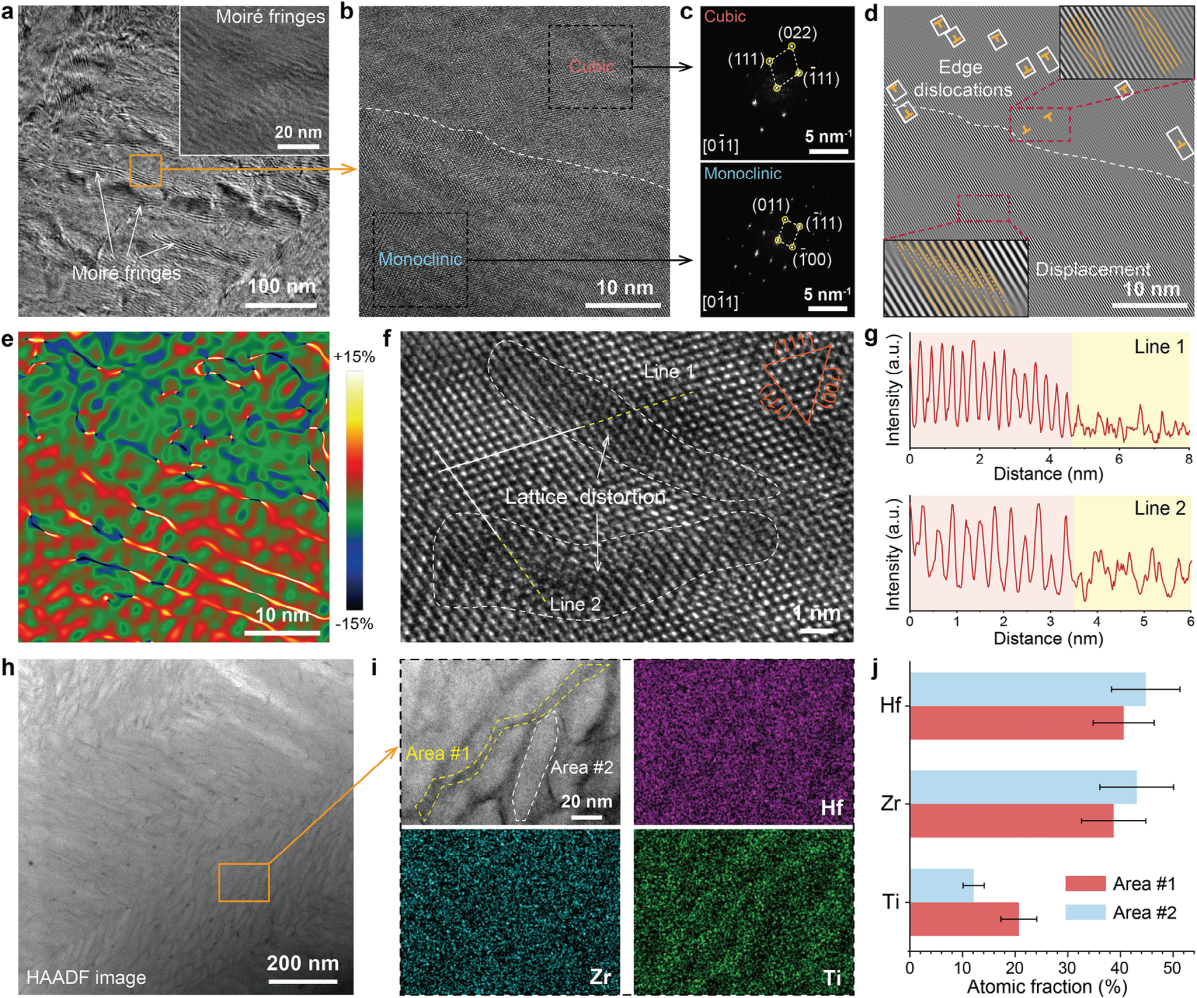
TEM analysis of the outer oxide scale (Ti‐rich layer) on the *x* = 0.1 coating after the 3^rd^ ablation cycle. a) BF‐TEM image and HR‐TEM image in the inset show moiré patterns. b) HR‐TEM image corresponding to the box in (a). c) Fast Fourier transform analysis determines the mixed phase composition of cubic and monoclinic structures. d) Inverse Fast Fourier transform analysis. e) The strain mapping corresponding to (b) through geometric phase analysis. f) Atomic‐resolution image of the monoclinic phase, where *d*_(100)_ = 5.078 Å and *d*_(111)_ = 2.825 Å. g) Intensity profiles along lines 1 and 2 in (f). The yellow region indicates severe lattice distortion. h,i) HAADF images and EDS elemental maps show Ti elemental segregation. j) EDS quantification corresponding to (i).

The outer layer acts as a satisfactory barrier against oxygen diffusion inward and hot gas scour due to fewer cracks considered fast oxygen pathways (Figure 3c). More interesting, the SEM image of the outer oxide scale formed after the 3^rd^ ablation cycle shows crack deflection, bridging, and thus termination (Figure S10, Supporting Information), indicating the toughness improvement. To elucidate the underlying mechanism potentially associated with Ti solid solution behaviors, TEM analyses on the outer layer of the oxide scale after 3^rd^ ablation cycle were carried out. The BF image in Figure S11a (Supporting Information) verifies the absence of micro‐cracks. More interestingly, the magnified BF image (**Figure** **6**a) and HR‐TEM image in the inset show moiré fringes, generally resulting from two similar patterns overlapping with different spacings or orientations. Figure 6b shows the HR‐TEM image of two regions with and without moiré patterns, the two areas being separated by a white dashed line. The fast Fourier transform analysis based on the HR‐TEM image (Figure 6c) reveals the m‐phase plus a special c‐phase. The c‐phase oxide coincides with the minor phase in the XRD results shown in Figure 3a and Figure S3 (Supporting Information). The SAED pattern (Figure S11b, Supporting Information) is consistent with the Fourier analysis results, exhibiting an overlay of two sets of diffraction spots (m‐phase plus c‐phase), as depicted in the inset. Figure 6d is the corresponding inverse fast Fourier transform image of Figure 6b with a normal crystallographic direction of the lattice planes ($\bar{1}$11). Notably, as depicted in the inset, c‐(Hf,Zr,Ti)O_2_ possesses a high dislocation density, and m‐(Hf,Zr,Ti)O_2_ exhibits a slight slip, producing the moiré fringes. Incorporating trivalent cations into Hf/ZrO_2_ to stabilize the c‐phase has been an implemented strategy. The underlying mechanism is that the host cations are trapped by the surrounding oxygen vacancies.^[^ ^55^, ^56^ ^]^ Interestingly, we demonstrate that Ti ions doping is also effective in partially stabilizing (Hf,Zr)O_2_ under oxyacetylene flame conditions. It is reasonable because the typical Ti‐oxide, TiO_2_, is a nonstoichiometric compound with predominant defects including interstitial Ti ions and oxygen vacancies.^[^ ^57^ ^]^ The Ti‐induced oxygen vacancies trapped partial cations in (Hf,Zr,Ti)O_2_ and thus retained the high‐temperature c‐phase. The metastable c‐phase is regarded as a toughener. It transforms into t‐phase and thus m‐phase under the stress field, accompanied by volume expansion, thereby forming compressive stress on the potential cracks, hindering crack propagation.^[^ ^58^ ^]^

It has been accepted that the strain field resulting from multicomponent solid solution also plays a role in toughening the oxide ceramics.^[^ ^53^ ^]^ Geometric phase analysis was performed on the HR‐TEM images (Figure 6b) to obtain the strain distribution. As shown in the strain mapping (Figure 6e), numerous isolated edge dislocations represented by symmetrical compression‐tensile strain pairs are observed in the c‐(Hf,Zr,Ti)O_2_ region, which probably associated with the existence of interstitial Ti ions. For the m‐(Hf,Zr,Ti)O_2_ region, a periodic strain field associated with slip exists, corresponding to the moiré pattern. Figure 6f shows the atomic‐resolution image of the m‐oxide, where *d*_(100)_ = 5.078 Å and *d*_(111)_ = 2.825 Å, consistent with that of monoclinic Zr/HfO_2_. The intensity profiles along lines 1 and 2 (Figure 6g) indicate local severe lattice distortion (see yellow regions on the right), which results in a large strain field. The HAADF image of the inner oxide scale and the corresponding EDS elemental maps (Figure 6h,i) show Ti elemental segregation at the nanoscale. According to the EDS quantification (Figure 6j), the Ti solubility in the Ti segregated and other areas is ≈20 mol% and ≈11 mol% respectively. The Ti segregation was responsible for the local severe lattice distortion shown in Figure 6f. The Ti segregation plus the intrinsic interstitial Ti ions, results in severe lattice distortion, contributing to the enlarged lattice strain illustrated in Figure 6e. The interaction between the large strain field and cracks renders crack deflection and bridging, enhancing the toughness of the oxide scale.

The unique Ti ion dissolving into the outer (Hf,Zr,Ti)O_2_ resulted in both the metastable c‐(Hf,Zr,Ti)O_2_ and the large strain field, enabling the toughness enhancement of the oxide scale. Therefore, fewer cracks are observed on the surface of the *x* = 0.1 oxide scale after the 120‐s ablation (Figure 3c), whereas intergranular and even intragranular cracks appear on the surface of the Ti‐free oxide scale (Figure 3b). The associated crack bridging, deflection, and thus termination observed on the outer *x* = 0.1 oxide scale after the three ablation cycles (Figure S10a,b, Supporting Information), are conducive to cycling reliability because the cracks are channels for rapid oxygen transport. Notably, the formation of the defective structure in the outer oxide scale essentially originates from the Ti‐related intrinsic defects, rather than from the defective structure induced by phase transformation. Additionally, compared with obtaining ultra‐dense dislocations through entropy engineering,^[^ ^53^ ^]^ incorporating an appropriate amount of Ti in the typical UHTCs is more economical and feasible, preventing elements that are probably detrimental to ablation resistance.

## 3 Conclusion

We fabricated multicomponent (Hf,Zr,Ti)B_2_ coatings on C/C composites by supersonic atmospheric plasma spraying, and investigated the effects of Ti content on the ablation performance under an oxyacetylene flame with a heat flux of 2.4 MW m^−2^. The (Hf_0.45_Zr_0.45_Ti_0.10_)B_2_ coating shows much better ablation resistance and cycling reliability (no ablation damage after three 120‐s ablation cycles; *R*_m_ = −0.18 mg s^−1^; *R*_l_ = −0.23 µm s^−1^) than the (Hf_0.50_Zr_0.50_)B_2_ and (Hf_0.40_Zr_0.40_Ti_0.20_)B_2_ coatings. The resulting functionally graded oxide scale, comprising an outer dense layer and an underlying fine granular layer, is responsible for the superior ablation performance. The analyses at the multi‐scale demonstrate that the unique Ti ion distribution in the inner and outer layers leads to the functionally graded architecture. Ti ions are uniformly distributed in the inner layer with a solubility of ≈4 mol%, rendering grain refinement via sluggish diffusion effect, and thus stress release. For the outer layer, more Ti dissolving induces metastable cubic (Hf,Zr,Ti)O_2_, leading to phase transformation toughening. The Ti segregation at the nanoscale results in local severe lattice distortion, inhibiting the propagation of cracks via the interaction between the huge strain field and cracks. Overall, the outer oxide scale with fewer cracks acts as a strong oxygen diffusion barrier, and the inner fine granular layer with high strain tolerance enables the oxide scale's structural integrity under severe thermal shock. This study provides new insights into the contribution of Ti doping to the oxide scale's ablation performance and guidance for further compositional designing of emerging multicomponent UHTCs for ultra‐high‐temperature applications.

## 4 Experimental Section

### Material and Preparation

C/C composites were fabricated by isothermal chemical vapor infiltration. During the process, pyrolytic carbon was infiltrated into 2.5D needle‐punched carbon fiber preform until the density reached ≈1.75 g cm^−3^. The C/C substrates (Φ30 × 5 mm) were machined from the C/C composite bulk, and then a SiC coating was first grown on the substrate by pack cementation to alleviate the thermal expansion mismatch between the substrate and the boride coating. Details of this process were available elsewhere.^[^ ^10^ ^]^ The preparation of the outer coatings starts with the boride powder synthesis by boro‐carbothermal reduction of transition oxides, which was a favorable process due to the fine grain of the products and few residual oxides.^[^ ^19^ ^]^ Taking the (Hf_0.5‐_ *_x_*_/2_Zr_0.5‐_ *_x_*_/2_Ti*_x_*)B_2_ (*x* = 0.20) powder as an example, the reduction process was dominated by Equation (1):

(1) $$\begin{array}{c} 2/5\mathrm{Hf}O_{2}+2/5\mathrm{Zr}O_{2}+1/5\mathrm{Ti}O_{2}+5/7B_{4}C=\left(Hf_{0.40}Zr_{0.40}Ti_{0.20}\right)B_{2}\\ +3/7B_{2}O_{3}\left(g\right)+5/7\mathrm{CO}\left(g\right) \end{array}$$

Based on Equation (1), commercially available HfO_2_, ZrO_2_, TiO_2_ powders (>99.9%, 1–3 µm; Qinhuangdao Eno Material Development Co. Ltd, China), and B_4_C powder (>99%, ≈3 µm; Qinhuangdao Eno Material Development Co. Ltd, China) were mixed. The molar amount of added B_4_C was increased by 15% to compensate for the additional loss of B‐species during heat treatment.^[^ ^59^ ^]^ The powder batches were homogenized using a planetary ball mill for 4 h under wet conditions of industrial alcohol, with Y_2_O_3_‐stabilized zirconia milling media. The mixtures were dried in an oven at 70 °C for 8 h and then passed through a 50‐mesh sieve. The mixtures were placed in graphite crucibles lined with graphite papers. The graphite crucibles were heated by an induction heating furnace under a flowing argon atmosphere. The temperature in the furnace was raised to 1800 °C at a heating rate of 5 °C min^−1^, with 2 h isothermal dwell. The obtained powders were used for SAPS after spray granulation. The crushed powder was mixed with alcohol, distilled water, and polyvinyl alcohol aqueous solution (0.02 g ml^−1^) according to the mass ratio of 4:1:1:4 by the planetary ball mill for 4 h. The obtained suspension was ejected into a drying tower where the inlet and outlet temperatures were ≈320 and ≈110 °C, respectively. The dried powder was passed through a 200‐mesh sieve and was used as feedstock for SAPS. The boride coatings were deposited onto the SiC‐coated C/C composites by a SAPS system. Details of the SAPS parameters are summarized in Table S2 (Supporting Information).

### Ablation Tests

The ablation performance of the UHTC boride coatings on C/C composite substrates was evaluated by the oxyacetylene flame ejected by a superalloy gun with a heat flux of 2.4 MW m^−2^. The flow rates of acetylene and oxygen were 0.18 L s^−1^ and 0.24 L s^−1^, respectively. The distance between the gun nozzle and the initial surface of the specimen was 10 mm. The surface center response temperature of the ablated center during the test was recorded by an infrared pyrometer (MR1SCSF, Raytek, USA). The three coatings were exposed to the flame for 120s duration, and the (Hf_0.5‐_ *_x_*_/2_Zr_0.5‐_ *_x_*_/2_Ti*_x_*)B_2_ (*x* = 0.10) coating was further subjected to three 120‐s ablation cycles, with a 5‐s interval of natural cooling between each cycle. The mass (*R*_m_) and linear (*R*_l_) recession rates were defined to assess the ablation performance of the samples calculated according to *R*_m_ = (*m*_0_‐*m*)/*t* and *R*_l_ = (*l*_0_‐*l*)/*t*, respectively, where *l*_0_ and *l* refer to the thickness of the specimen before and after the torch testing, respectively; *m*_0_ and *m* represent the mass of the sample before and after the torch testing, respectively; *t* is the ablation duration. The thickness was measured at the severely heat‐affected zone, and the average value of three measurements was finally adopted.

### Characterizations

The phase composition of the samples was identified by XRD (X'Pert Pro MPD, Panalytical, Netherlands) using Cu Kα radiation with a step size of 0.02° and 1 s dwell counting time. The SEM images and corresponding elemental analyses were conducted by a scanning electron microscope (EVO10, Zeiss, Germany) operated at a 20 kV accelerating voltage. The cross‐sectional elemental distribution of the coating after ablation testing was analyzed by wavelength dispersive spectroscopy in an electron probe microanalyzer (EPMA‐8050G, Shimadzu, Japan), which allows the analysis of light elements like B and O. The BF images, HR‐TEM images, SAED patterns, HAADF images, and elemental analysis were obtained by an aberration‐corrected (scanning) transmission electron microscope (Themis Z, Thermo Fisher Scientific; USA) operated at 200 kV. The TEM specimens were extracted from the outer and inner layers of the oxide scale on the coating after the 3^rd^ ablation testing by an in situ lift‐out technique using a focus ion beam microscope (Helios G4 CX, Thermo Fisher Scientific, USA). The particle size distributions were counted by Nano Measure software.

## Conflict of Interest

The authors declare no conflict of interest.

## Supporting information



Supporting Information

## Data Availability

The data that support the findings of this study are available from the corresponding author upon reasonable request.
