# Supplementary material for: Functionally Graded Oxide Scale on (Hf,Zr,Ti)B2 Coating with Exceptional Ablation Resistance Induced by Unique Ti Dissolving
Source: Adv Sci (Weinh). 2025 Jan 15;12(10):2411292. doi: 10.1002/advs.202411292 (PMC11904937; doi:10.1002/advs.202411292)
Supplement: Supplementary file 1 — Supporting Information [file ADVS-12-2411292-s001.pdf]

ADVANCED SCIENCE
Open Access

Supporting Information

for *Adv. Sci.*, DOI 10.1002/advs.202411292

Functionally Graded Oxide Scale on (Hf,Zr,Ti)B_2 Coating with Exceptional Ablation Resistance Induced by Unique Ti Dissolving

Junshuai Lv, Wei Li, Yanqin Fu, Menglin Zhang, Lingxiang Guo, Fanyu Lu, Jiachen Li, Tao Li, Yulei Zhang* and Hejun Li*

Supporting Information for

Functionally Graded Oxide Scale on $(Hf,Zr,Ti)B_2$ Coating with Exceptional Ablation Resistance Induced by Unique Ti Dissolving

Junshuai Lv [a], Wei Li [a], Yanqin Fu [b], Menglin Zhang [a], Lingxiang Guo [a], Fanyu Lu [a], Jiachen Li [a], Tao Li [b,*], Yulei Zhang [a,b,*], Hejun Li [a]

[a] *Shaanxi Key Laboratory of Fiber Reinforced Light-Weight Composites, State Key Laboratory of Solidification Processing, Northwestern Polytechnical University, Xi'an 710072, China*

[b] *Henan Key Laboratory of High Performance Carbon Fiber Reinforced Composites, Institute of Carbon Matrix Composites, Henan Academy of Sciences, Zhengzhou 450046, China*

[*]Corresponding author

E-mail address: zhangyulei@nwpu.edu.cn (Yulei Zhang); Tel: +86 371 65738933; Fax: +86 371 65722960.

E-mail address: litao@hnas.ac.cn (Tao Li).

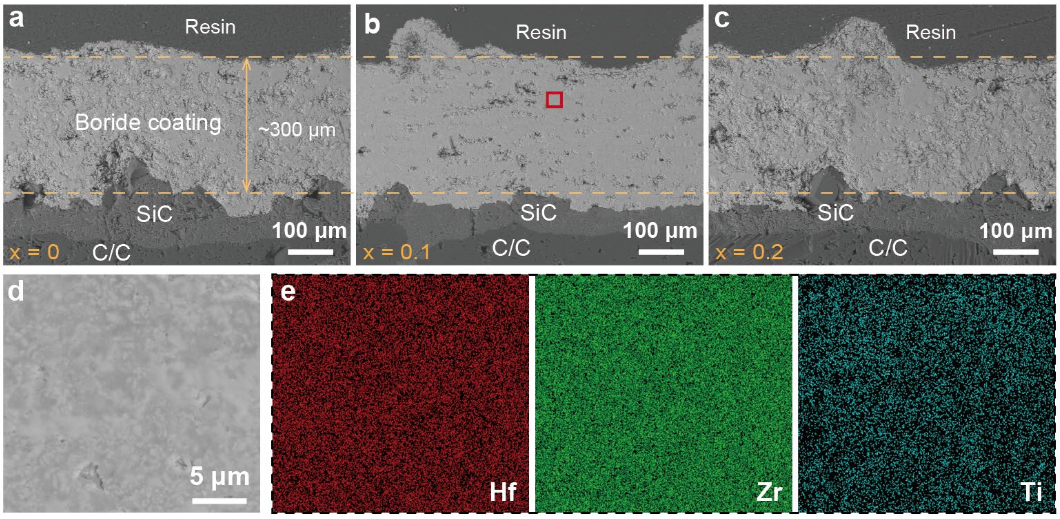

Figure S1 a-c) Cross-sectional SEM images of the $(Hf_{0.50\text{-}x/2}Zr_{0.50\text{-}x/2}Ti_{x})B_2$ (x = 0, 0.10, and 0.20) coatings show coatings' average thicknesses of about 300 μm. d) Magnified SEM image inside the red box in b). e) EDS elemental maps corresponding to d) show uniform elemental distribution.

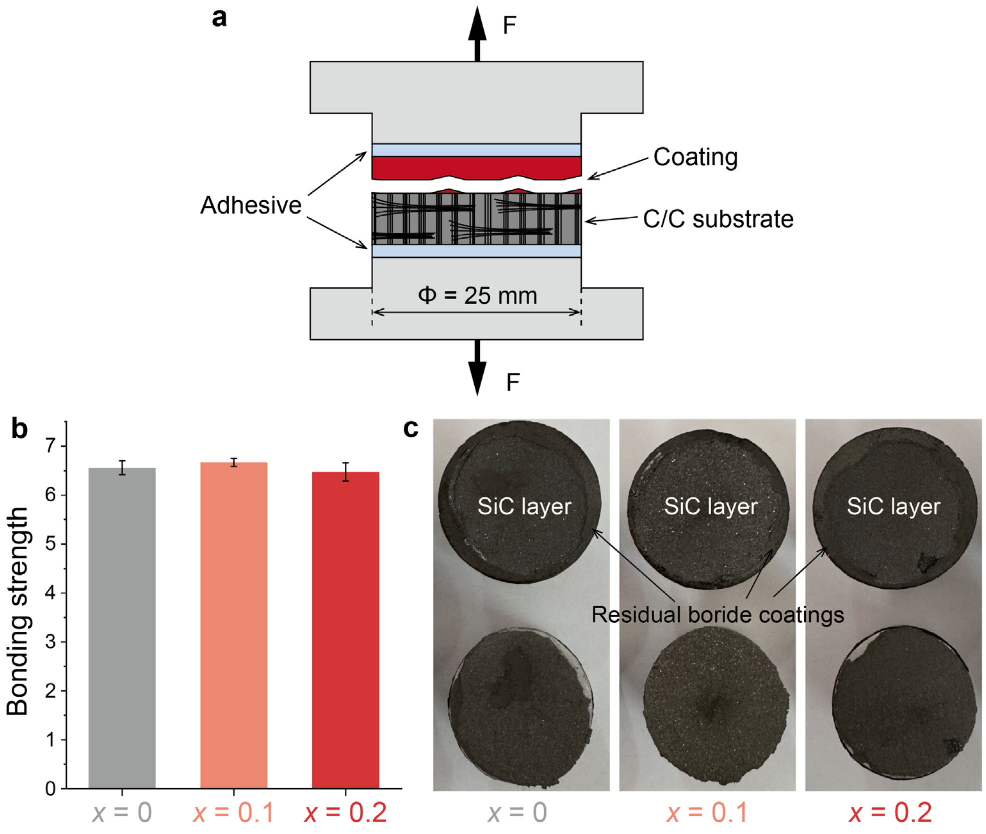

Figure S2 a) Illustration of the coating bonding strength test. b) Bonding strength of the sprayed boride coatings. c) Optical images of the tensile fracture surfaces on the pairs.

The coatings' bonding strengths were measured *via* tensile tests, as illustrated in **Figure S2a**. The samples were adhered to aluminum alloy pairs with epoxy resin, with the top and bottom adjusted to be coaxial. They were then placed at room temperature for 48 hours to ensure the resin was cured. Finally, they were mounted on a mechanical testing machine and pulled to fracture at a loading rate of 0.05 mm/s. The bonding strength (σ) was calculated as $\sigma = F_{max}/A$, where F_{max} is the maximum applied load and A is the circular contact area with a diameter of 25 mm. The bonding strength of each coating is the average of three measurements. **Figure S2b** shows similar bonding strengths of the coatings (~6.5 MPa), suggesting it is independent of the coatings' composition. All the coatings were debonded at the interface between the sprayed boride and SiC layers.

Table S1

Details of the ablation performance of the $(Hf_{0.45}Zr_{0.45}Ti_{0.10})B_2$ coating compared with representative modified Hf/ZrB_2 coatings and multicomponent UHTC coatings.

	Process	Temperature (°C)	Duration (s)	R_l (μm s^{-1})	R_m (mg s^{-1})	Ref.
$(Hf_{0.45}Zr_{0.45}Ti_{0.10})B_2$	SAPS	~2200	120	-0.53	-0.63	This work
$(Hf_{0.45}Zr_{0.45}Ti_{0.10})B_2$	SAPS	~2200	360	-0.23	-0.18	This work
HfB_2	SAPS	~1900	30	-0.32	-0.14	[1]
HfB_2-SiC	GSI	~2000	180	1.62	/	[2]
ZrB_2-$MoSi_2$	SAPS	~1800	30	1.67	0.44	[3]
ZrB_2-La_2O_3	SAPS	~2100	120	-0.017	−0.56	[4]
HfB_2-SiC-Si	SD	~2000	90	0.31	-0.36	[5]
HfB_2-WSi_2-SiC	PC	/	90	-0.39	0.068	[6]
HfB_2-$CrSi_2$-SiC	PC	~1800	90	-0.42	-0.046	[7]
ZrB_2-$CrSi_2$-Si	PC	~2300	90	1.64	0.56	[8]
ZrB_2-$TiSi_2$-SiC	SAPS	~1957	240	0.22	0.44	[9]
ZrB_2-SiC-ZrC	SAPS	~2300	200	-0.065	−0.23	[10]
$(Zr_{0.5}Ta_{0.5})B_2$-Si-SiC	SD	~1600	60	-0.35	-0.15	[11]
$(Zr_{0.7}Ta_{0.3})B_2$-SiC	SD	~2300	120	3.01	-0.033	[12]
$(Hf,Zr,Ti,Cr)B_2$-SiC-Si	GSI	~2130	60	-0.15	0.37	[13]
$(Hf_{0.50}Zr_{0.50})B_2$-SmB_6-ErB_6-YB_6	SAPS	~2319	60	0.19	0.33	[14]
$(Hf,Zr,Ta,Ti)B_2$	SAPS	~1990	60	1.14	-0.63	[15]
$(Hf,Zr,Ta)B_2$	SAPS	~2160	240	0.11	-0.47	[16]
HfC-ZrC-TiC	SAPS	~2100	120	-0.71	-0.18	[17]
(Hf,Zr,Ti)C	SAPS	~2168	210	-0.15	-0.19	[18]
$(Hf_{0.25}Zr_{0.25}Ti_{0.5})C$	SAPS	~2100	120	0.67	−0.82	[19]

SAPS: Supersonic atmospheric plasma spraying; GSI: Gasous silicon infiltration; SD: Slurry dipping; PC: Pack cementation.

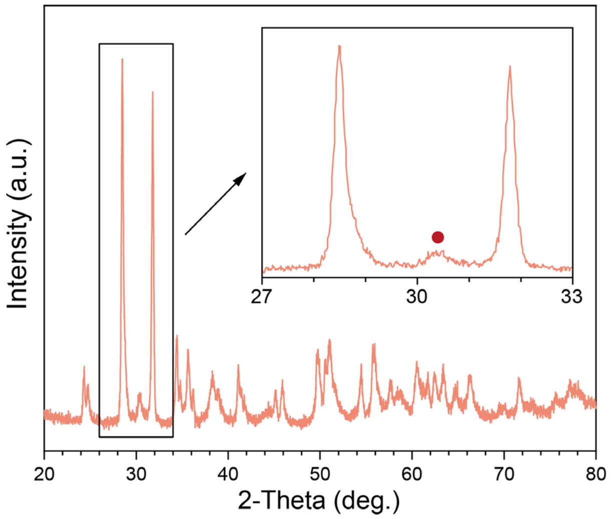

Figure S3 XRD pattern on the surface center of the $x = 0.1$ coating's oxide scale formed after three 120-s ablation cycles reveals the existence of the minor phase.

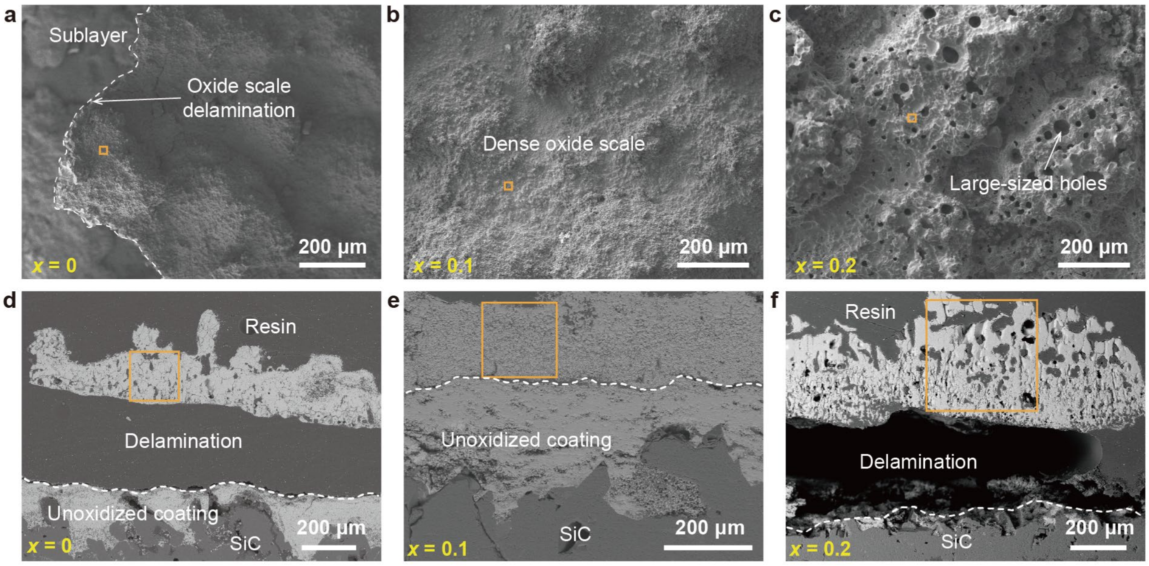

Figure S4 Surface and cross-sectional SEM images of the $(Hf_{0.50-x/2}Zr_{0.50-x/2}Ti_x)B_2$ (x = 0, 0.10, and 0.20) coatings after 120-s ablation testing. a,d) x = 0. b,e) x = 0.10. c,f) x = 0.20. The areas within the boxes are consistent with the magnified images shown in Figure 3, respectively.

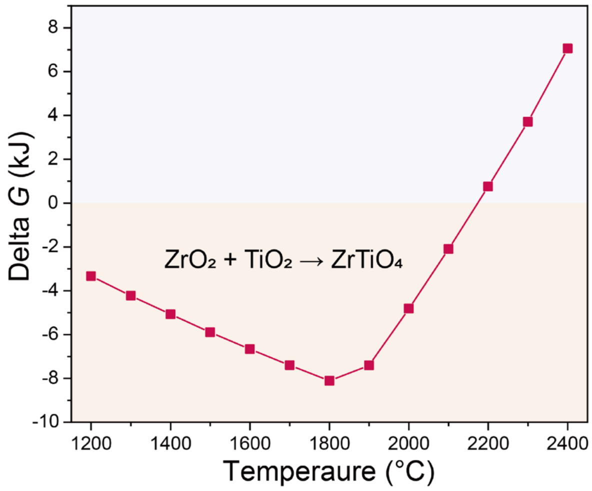

Figure S5 Δ*G* of the reaction: $ZrO_2 + TiO_2 \rightarrow$ orthorhombic-$ZrTiO_4$ (Δ*G* was calculated by HSC chemestry 6.0 software).

Taking an example of the reaction to form orthorhombic-$ZrTiO_4$, its Δ*G* is a positive value at the response temperature (~2200°C), thus implying its instability. In other words, the composition of the oxide scale during the temperature-stabilized ablation stage did not include (Hf,Zr)TiO_4, but rather a tetragonal-(Hf,Zr,Ti)O_2 solid solution plus Ti-rich liquid theoretically.

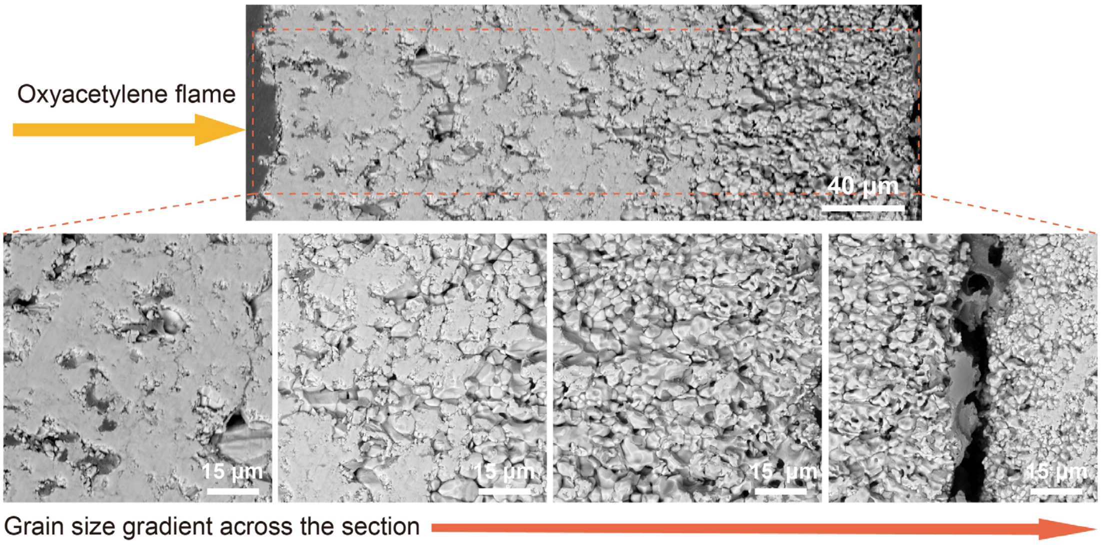

Figure S6 Magnified cross-sectional SEM images of the resulting $x = 0.1$ oxide scale after the 3rd ablation cycle showing a grain size gradient across the section.

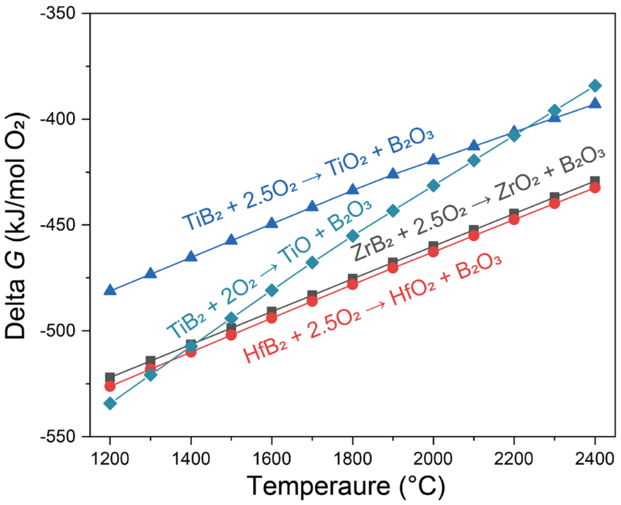

Figure S7 ΔG of the oxidation reactions of MB_2 (M = Hf, Zr, and Ti) calculated by HSC chemestry 6.0 software.

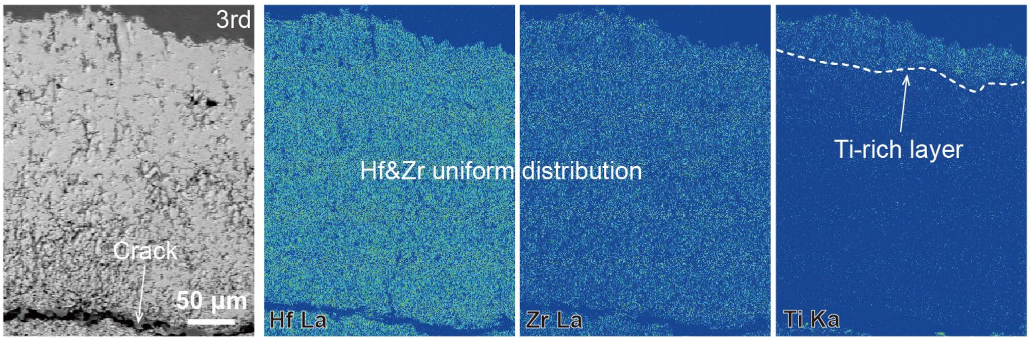

Figure S8 Overviews of the oxide scales with EPMA elemental maps of Hf, Zr, and Ti forming on the $(Hf_{1-x/2}Zr_{1-x/2}Ti_x)B_2$ (x = 0.1) coating after the 3rd 120-s ablation cycle.

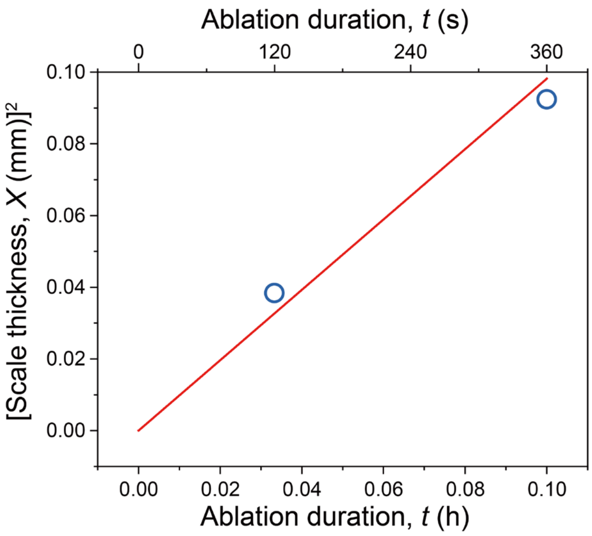

Figure S9 Parabolic rate constant fitting for oxide growth (k_p = 1.06 ± 0.04 mm^2/h) on the x = 0.1 coating.

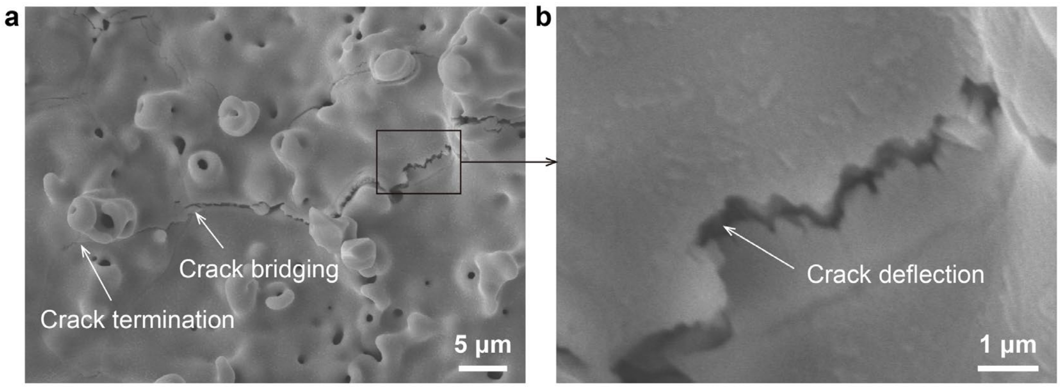

Figure S10 a) Surface SEM image of the resulting x = 0.1 oxide scale after the 3rd ablation cycle showing crack deflection, bridging, and termination. b) Magnified image inside the box in a).

Crack deflection, bridging, and termination are observed on the oxide scale formed after the 3rd ablation test, which can be considered as evidence of improvement in toughness, preventing drastic cracking of the oxide scale. Fewer cracks also contribute to improved ablation resistance because they are channels for rapid oxygen transport.

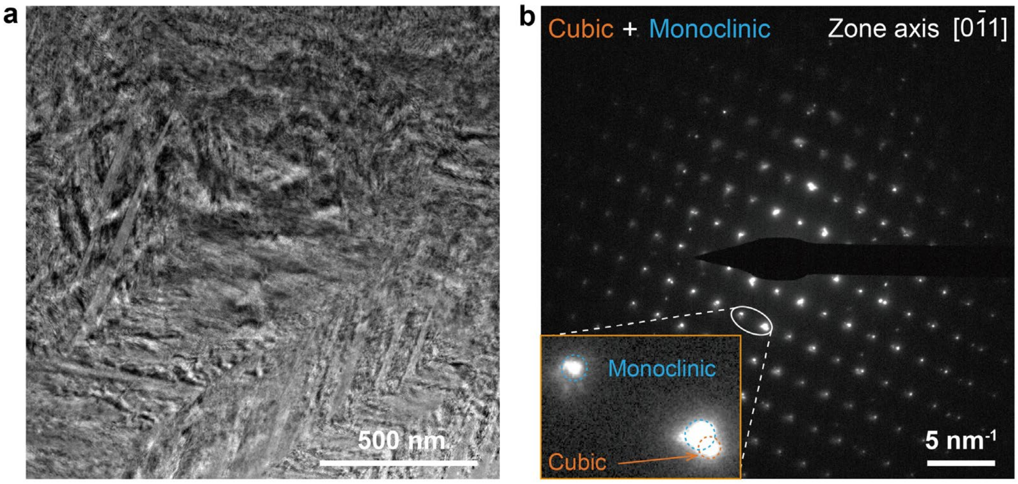

Figure S11 a) BF-TEM image of the dense top layer of the x = 0.1 oxide scale after the 3rd ablation cycle showing the absence of cracks. b) SAED pattern showing overlay of two sets of diffraction spots (m-phase plus c-phase).

Table S2 Parameters of the SAPS system for deposition of the (Hf,Zr,Ti)B_2 coatings.

Parameters	Setting
Spray current (A)	400–420
Torch power (kW)	49–51
Primary gas Ar (L/min)	73–76
Second gas H_2 (L/min)	5–15
Carrier gas Ar (L/min)	9–12
Feed rate (g/min)	25
Spray distance (mm)	100

Supplementary References

[1] K. Li, G. Liu, Y. Zhang, *Surf. Coat. Technol.* **2019**, 357, 48-56.

[2] L. Zhou, J. Zhang, D. Hu, Q. Fu, W. Ding, J. Hou, B. Liu, M. Tong, *J. Mater. Sci. Technol.* **2022**, 111, 88-98.

[3] D. Hu, Q. Fu, T. Liu, M. Tong, *J. Eur. Ceram. Soc.* **2020**, 40, 212-219.

[4] M. Chen, X. Yao, G. Feng, Y. Guo, *Ceram. Int.* **2020**, 46, 28758-28766.

[5] Y. Jiang, T. Liu, H. Ru, W. Wang, C. Zhang, X. Yue, *J. Alloys Compd.* **2019**, 782, 761-771.

[6] P. Wang, H. Li, R. Yuan, W. Xie, Y. Zhang, *Corros. Sci.* **2020**, 177, 108964.

[7] P. Wang, H. Li, R. Yuan, J. a. Kong, T. Li, Y. Zhang, *Corros. Sci.* **2020**, 167, 108536.

[8] T. Feng, H. Li, M. Hu, H. Lin, L. Li, *J. Alloys Compd.* **2016**, 662, 302-307.

[9] J. Li, Y. Zhang, H. Wang, Y. Fu, G. Chen, Z. Xi, *J. Alloys Compd.* **2020**, 824, 153934.

[10] Y. Zhang, H. Hu, P. Zhang, Z. Hu, H. Li, L. Zhang, *Surf. Coat. Technol.* **2016**, 300, 1-9.

[11] Y. Jiang, S. Yin, M. Li, Z. Zhang, G. Tang, N. Wang, H. Ru, *Ceram. Int.* **2021**, 47, 11358-11371.

[12] K. Tong, M. Zhang, Z. Su, X. Wu, C. Zeng, X. Xie, C. Fang, C. Yang, Q. Huang, D. Huang, *Corros. Sci.* **2021**, 188, 109545.

[13] P. Zhang, C. Cheng, M. Xu, B. Liu, X. Zhu, Q. Fu, *Ceram. Int.* **2022**, 48, 27106-27119.

[14] L. Guo, Y. Wang, B. Liu, Y. Zhang, Y. Tang, H. Li, J. Sun, *J. Eur. Ceram. Soc.* **2023**, 43, 1322-1333.

[15] J. Lv, W. Li, T. Li, L. Guo, Y. Fu, J. Li, J. Zhang, Y. Zhang, *Compos. Part B-Eng.* **2024**, 270, 111137.

[16] J. Lv, W. Li, T. Li, B. Gao, J. Li, Y. Fu, L. Guo, Y. Zhang, *J. Mater. Sci. Technol.* **2025**, 204, 115-126.

[17] J. Li, Y. Zhang, J. Lv, T. Li, X. Zhu, W. Gai, *Corros. Sci.* **2022**, 205, 110474.

[18] J. Li, Y. Zhang, Y. Zhao, Y. Zou, J. Lv, J. Li, *Compos. Part B-Eng.* **2023**, 251, 110467.

[19] J. Li, F. Lu, T. Li, J. Lv, F. Zhu, Y. Zhang, *Surf. Coat. Technol.* **2024**, 493, 131293.
